# Point‐of‐care detection of cytokines in cytokine storm management and beyond: Significance and challenges

**DOI:** 10.1002/VIW.20210003

**Published:** 2021-05-04

**Authors:** Guozhen Liu, Cheng Jiang, Xiaoting Lin, Yang Yang

**Affiliations:** ^1^ School of Life and Health Sciences The Chinese University of Hong Kong Shenzhen 518172 P.R. China; ^2^ Graduate School of Biomedical Engineering University of New South Wales Sydney NSW 2052 Australia; ^3^ Nuffield Department of Clinical Neurosciences John Radcliffe Hospital University of Oxford Oxford OX3 9DU United Kingdom

**Keywords:** COVID‐19, cytokine biosensors, cytokine storm, disposable sensors, point‐of‐care diagnostics

## Abstract

Cytokines are signaling molecules between cells in immune system. Cytokine storm, due to the sudden acute increase in levels of pro‐inflammatory circulating cytokines, can result in disease severity and major‐organ damage. Thus, there is urgent need to develop rapid, sensitive, and specific methods for monitoring of cytokines in biology and medicine. Undoubtedly, point‐of‐care testing (POCT) will provide clinical significance in disease early diagnosis, management, and prevention. This review aims to summarize and discuss the latest technologies for detection of cytokines with a focus on POCT. The overview of diseases resulting from imbalanced cytokine levels, such as COVID‐19, sepsis and other cytokine release syndromes are presented. The clinical cut‐off levels of cytokine as biomarkers for different diseases are summarized. The challenges and perspectives on the development of cytokine POCT devices are also proposed and discussed. Cytokine POCT devices are expected to be the ongoing spotlight of disease management and prevention during COVID‐19 pandemic and also the post COVID‐19 pandemic era.

## Graphical abstract

Due to the significance of cytokines in biology and medicine, great advances have been witnessed with respect to cytokine biosensing technologies and cytokines clinical utility. With the aid of digital monitoring tools, POC detection of cytokines as biomarkers might step into new era toward disease diagnosis, prevention and management.

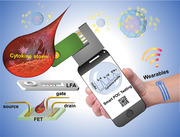

## 1 INTRODUCTION

The emerging aggressive COVID‐19 pandemic is continuing to challenge medical health systems all over the world.  Cytokine storm in COVID‐19 results from a sudden acute increase in circulating levels of different pro‐inflammatory cytokines and can cause several disease and major‐organ injury.^[^ ^1^, ^2^ ^]^ Early diagnosis of COVID‐19 virus infection is helpful to monitor and track the spread of COVID‐19.^[^ ^3^ ^]^ Cytokine monitoring is under the spotlight of disease prevention and protection due to the continuous threaten of COVID‐19 and biological significance of cytokines.^[^ ^3^ ^]^ The early recognition of cytokine storm by monitoring cytokine levels and the prompt interventions may halt progression to severe/critical disease and lead to better outcomes. The US Food and Drug Administration (FDA) has authorized blood purification product for reducing the amount of cytokines in the bloodstream that control immune response by filtering the blood and returning the filtered blood to the COVID‐19 patient suffered from “cytokine storm”.^[^ ^4^ ^]^ Therefore, it is essential to monitor treatment outcome by the detection of the blood cytokines.

Cytokines are glycoproteins with molecular weight ranging from about 6 to 70 kDa, which are mainly secreted by immune cells such as leukocytes, lymphocytes (T cells) and epithelial cells.^[^ ^5^ ^]^ Cytokines synchronize immune system responses with either pro‐inflammatory effect, anti‐inflammatory effect or contextual effect.^[^ ^6^ ^]^ According to their cell of origin or their mechanism of action, cytokines include interleukins, lymphokines, monokines, interferons, colony stimulating factors, etc., which also can be grouped as pro‐inflammatory cytokines and anti‐inflammatory cytokines. Cytokines can form a very complex cytokine network, and numerous cytokines are pleiotropic effectors showing multiple biological activities. Cytokines, as immunomodulation agents have pivotal significance in biology and medicine, and have doubled‐edge sworn function to our health.^[^ ^7^ ^]^ Cytokines on one hand they are critical to eliminate the infection while on the other, excessive production can cause tissue and organ damage due to the over response of immune system.^[^ ^7^ ^]^ The immune imbalance and the cytokine dysregulation can result in diseases and unhealthy conditions. Thus, there are unmet demands for having the sensitive, reliable, and rapid methods for screening of cytokine secretions to understand the for precise early diagnosis of diseases, disease management, and interventions in severe pathophysiological conditions.^[^ ^8^, ^9^ ^]^

Point‐of‐Care (POC) or Point‐of‐Need testing, as tests can be conducted at or near patients’ (end‐users’) site, has attracted dramatic attentions in various areas such as health care, environmental and food safety by providing a quick, simple, and cost‐effective way of detection.^[^ ^10^, ^11^ ^]^ Ideal POCT meet the ASSURED (affordable, sensitive, specific, user‐friendly, rapid and robust, equipment‐free, and deliverable to end users) criteria, outlined by the World Health Organization. They are superior to the traditional laboratory‐based detection methods such as polymerase chain reaction (PCR) at messenger RNA levels^[^ ^12^ ^]^ and enzyme‐linked immunosorbent assay (ELISA) kits at protein levels.^[^ ^13^ ^]^ Unfortunately, few studies are reported on the POC detection of cytokines because of challenges associated with cytokine detection such as low concentration, thermal instability of cytokines, dynamic secretion process and complex cytokine network, etc.^[^ ^8^ ^]^ This review aims to summarize the biological significance of cytokines and roadmap of cytokine detection in COVID‐19 and other diseases. The clinical cut‐off levels of cytokines as biomarkers for different diseases are summarized. Different strategies for POC detection of cytokines are highlighted. Challenges and future perspectives on cytokine biomarker development and their translation toward routine POC diagnostics are proposed.

## 2 BIOLOGICAL SIGNIFICANCE OF CYTOKINES

### 2.1 Double‐edged sword function in health care

Cytokines, signaling molecules between cells, medicate and regulate immunity, inflammation and haematopoiesis, and thus they are indicators of body health conditions.^[^ ^14^, ^15^ ^]^ The understanding of the cytokine secretions provides medical knowledge on mechanisms of pathologies and to the development of new treatments with biological drugs. Cytokine levels elevate during the course of diseases like rheumatoid arthritis, cardiovascular and neurodegenerative disease, sepsis, diabetes, and cancers, making them potential biomarkers for many diseases.^[^ ^16^, ^17^, ^18^, ^19^, ^20^, ^21^, ^22^ ^]^ For example, cytokine INF‐γ secreted by T‐cells has functioned as a biomarker for the diagnosis of tuberculosis (TB).^[^ ^23^ ^]^ A very decent performance with overall sensitivity of 85.5% and specificity of 97.7% was achieved using blood samples from a cohort with 83 patients and 43 healthy controls (HC). Another recent study shows that a combination of IFN‐γ, IP‐10, ferritin and 25 hydroxyvitamin D has potential for the diagnosis of pediatric TB and discrimination between TB and latent TB infection (LTBI) in a recruited group of 166 children (74 with active TB, 37 with LTBI, and 55 uninfected controls).^[^ ^24^ ^]^ In their characteristic (ROC) curve model, an area under curve (AUC) of 0.955 with an optimal sensitivity (93.2%) and specificity (90.0%), indicating its high diagnostic accuracy for stepping into next phase with larger cohort validation and clinical practice. Moreover, increased levels of pro‐inflammatory cytokines, such as IL‐10, TGF‐β‐1, TNF‐α, IL‐1, and IL‐6, are believed to be implicated in the deterioration of heart failure because these cytokines impacted chronic kidney dysfunction and persistent congestion and consequently influenced heart failure prognosis, which is a global health issue causing a huge economic burden (estimated at $108B per annum).^[^ ^25^ ^]^ Results from experimental and clinical trials suggest that inflammatory mediators such as cytokines play an essential role in the pathogenesis of chronic heart failure by regulating cardiac function.^[^ ^26^ ^]^ Furthermore, as a chronic inflammatory disease of the gastrointestinal tract, inflammatory bowel diseases such as Crohn's disease and ulcerative colitis are results of the imbalance interactions between pro‐inflammatory and anti‐inflammatory cytokine network.^[^ ^27^ ^]^ Additionally, the aging and aging‐related diseases are also closely related to the immune imbalance and the cytokine dysregulation.^[^ ^28^, ^29^ ^]^ A recent study showed that senescent cells expressed increased levels of IL‐6 and other senescence‐associated secretory phenotype components such as MCP‐1, eotaxin, growth differentiation factor 15 (GDF‐15), and fibroblast growth factor (FGF) as revealed in mouse model.^[^ ^30^ ^]^ These studies on mouse model or clinical cohort have demonstrated that various types of cytokines have high correlation with specific diseases, which paves a good way for developing cytokine bioFco in clinical practice and also promoting the need for development of cytokine detection platforms.

As immune mediator, cytokines perform context‐dependent functions and can exert opposing effects depending on the stage of inflammation, with important implications in diseases diagnosis and management. For example, cytokines, pro‐ and anti‐inflammatory cytokines, play double‐edged sword function in the complex pathophysiology underlying sepsis.^[^ ^22^ ^]^ It was observed that the cytokine network of IL‐6, IL‐8, monocyte chemoattractant protein 1 (MCP‐1), and IL‐10 contributes to the acute phase of sepsis.^[^ ^31^ ^]^ Increase in levels of cytokines such as IL‐6, Il‐8, IL‐10, IL‐18, and TNF‐α may have implications in diagnosis and treatment of sepsis.^[^ ^7^ ^]^ Cytokines also demonstrated their dual roles in Alzheimer's disease.^[^ ^32^ ^]^ Interleukins, TNF‐α, TGF‐β, and IFN‐γ are believed to actively participate in Alzheimer's disease pathogenesis by impacting on the Alzheimer´s amyloid precursor protein to affect its expression levels and amyloidogenic processing and/or β‐amyloid aggregation.^[^ ^33^ ^]^ They may serve as diagnostic or therapeutic targets for Alzheimer's disease neurodegeneration. Recently, a case‐control study (72 Parkinson's disease patients, 56 HC) was reported with investigation of selected serum immune mediator such as cytokines (IFN‐γ, TNFα, and IL‐10) and nitric oxide (NO_x_) in Parkinson's disease progression.^[^ ^34^ ^]^ TNF‐α‐mediated neurotoxicity appears to occur in early Parkinson's disease (PD), but both IFN‐γ and IL‐10 are involved in disease severity. With NO_x_, these three serum cytokines can be potential multimarker biosignature panels for PD of varying durations. The combination of the three factors, that is, IFN‐γ, IL‐10, and NO_x_‐based composite maker pattern, showed very profound discrimination capability for early and late PD with sensitivity of 93.3%, specificity of 87.5%, and AUC of 0.924, respectively. Another independent study demonstrated that the plasma concentrations of TNF‐α, IL‐10, and IFN‐γ were significantly higher in PD patients than in control groups (*p *<  0.001), which were associated with specific changes in gut microbiota.^[^ ^35^ ^]^ It suggests the microbiota alterations in PD patients associated with aberrant host immune responses are linked with PD pathogenesis. Fecal metabolomic analysis suggested gut microbiota is linked to inflammation and proinflammatory cytokines, and gut microbiota may predict the predisposition of normal individuals to severe COVID‐19.^[^ ^36^ ^]^ Thus detection of cytokine in stool might be another way for PD or other inflammation‐related disease diagnosis and management.

### 2.2 Cytokine release syndrome

Cytokine release syndrome is caused by a large, rapid release of cytokines into the blood from immune cells affected by infections or immunotherapy, and can results in cytokine storm in which the immune system fails to control.^[^ ^37^, ^38^ ^]^ This situation makes the sensitive and rapid cytokine monitoring significantly essential. Currently, a consensus is that “cytokine storm” is responsible for the poor prognosis of critical COVID‐19 cases resulting in high morbidity and mortality.^[^ ^39^, ^40^ ^]^ Cytokines produced during COVID‐19 infection target in the chronic inflammatory diseases,^[^ ^41^ ^]^ and thus older adults and people with pre‐existing chronic conditions, such as diabetes, chronic obstructive pulmonary disease, and hypertension suffer more severe COVID‐19 outcomes.^[^ ^42^ ^]^ Gnjatic and his team proposed that serum IL‐6 and TNF‐α levels should be considered in the management and treatment of patients with COVID‐19 to stratify prospective clinical trials, guide resource allocation and inform therapeutic options. Increasing evidence demonstrated that cytokines have implications for disease progression. Abundant research has demonstrated that the particular cut‐offs of cytokines as biomarkers can potentially be used for disease diagnosis (Table 1). Symptoms of diseases (e.g., COVID‐19, sepsis, Alzheimer ’s disease, etc.) are the results of the synergic actions of multiple cytokines. Their elevation or attenuation across cohorts containing disease cases and healthy controls would help discover and validate the most‐relevant cytokines and their cut‐off values for the corresponding diseases. Moreover, the cut‐off criteria of same cytokines in same bodily fluids for a specific disease are variable across different cohort studies, which is mainly due to the variations in detection kits that used in different labs and the factor of ethnicity, population in the cohort design. This might be solved by using uniform “standard” kits or a reliable corrections among different kits with the support of meta‐analysis. As a result, no study has provided conclusive results indicating cytokines are biomarkers for these diseases. To our knowledge, no cytokine has been approved to be biomarker for COVID‐19 or any other specific disease by FDA yet. One healthy condition is normally the results of a group of cytokines. Considering the variable outcomes of single cytokine level, combinational quantification of multiple cytokines provides accurate and precise information for diseases diagnosis by providing a comprehensive picture on disease evolution and progression. It is expected that cytokines are continuing to be the rising stars in the fields of molecular diagnosis, disease early diagnosis, and immunotherapy with the advances in biomedical research and the aid of sensitive monitoring tools.

**TABLE 1:** Cytokines clinical cut‐off levels as biomarkers

		Cut‐off concentration (pg/mL)			
Cytokine	Disease	Predict/hospitalization	Death/Organ failure	Sample	Ref.
IL‐6	Covid‐19		37.65	serum	[43]
IL‐6	Covid‐19		80	Plasma	[44]
IL‐6	Covid‐19		86	Plasma	[45]
IL‐6	Covid‐19		163.4	Serum	[46]
IL‐6	Covid‐19	9.16		Serum	[47]
IL‐6	Tuberculosis	4000		Pleural fluid	[48]
IL‐6	Ventilator‐associated pneumonia	198		Serum	[49]
IL‐6	Neonatal sepsis	30		Whole blood	[50]
IL‐6	Neonatal sepsis	10.85	78.2	Whole blood	[51]
IL‐6	System lupus erythematosus	12.3		Serum	[52]
IL‐6	Surgical site infection (periprosthetic joint infection)	359.3		Synovial fluid	[53]
IL‐6	Spontaneous bacterial peritonitis	5050		Ascitic fluid	[54]
IL‐6	Spontaneous bacterial peritonitis	1800		Serum	[54]
IL‐6	Acute appendicitis (children)	4.3		Serum	[55]
IL‐6	Acute pancreatitis		122	Plasma	[56]
IL‐6	Intertrochanteric fractures (the aged)		79.50	Plasma	[57]
IL‐1β	Ventilator‐associated pneumonia	10		Serum	[49]
IL‐1β	Neonatal sepsis	1		Whole blood	[50]
IL‐1β	Surgical site infection (periprosthetic joint infection)	8.26		Synovial fluid	[58]
IL‐8	Ventilator‐associated pneumonia	2000		Serum	[49]
IL‐8	Neonatal sepsis	60		Whole blood	[59]
IL‐10	Neonatal sepsis	14		Whole blood	[51]
IL‐13	Alzheimer's Disease	9.315		Serum	[60]
TNF‐α	Spontaneous bacterial peritonitis	63		Ascitic fluid	[54]
TNF‐α	Spontaneous bacterial peritonitis	48		Serum	[54]
TNF‐α	Endometriosis (adolescents)	3		Peritoneal fluid	[61]
TNF‐α	Intertrochanteric fractures (the aged)		55.27	Plasma	[57]
TGF‐β	System lupus erythematosus	54.2		Serum	[62]
IFN‐γ	Mycobacterium tuberculosis	4000		Whole blood	[63]
IFN‐γ	Tuberculosis	2850		Whole blood	[64]
IFN‐γ	Tuberculosis	60		Pleural fluid	[48]
IFN‐γ	Tuberculosis	112		Peritoneal ascites	[48]
IFN‐γ	Surgical site infection (periprosthetic joint infection)	34		Synovial fluid	[65]
IP 10	Tuberculosis	350		Whole blood	[66]
IP 10	Alzheimer's Disease	53.65		Serum	[60]

IL‐6: Interleukin 6, TNF‐α: tumor necrosis factor alpha, TGF‐β: transforming growth factor β, IFN‐γ: interferon‐gamma, IP‐10: interferon gamma‐induced protein‐10, IL‐1β: interleukin 1β, IL‐8: interleukin 8, IL‐10: interleukin 10, IL‐13: interleukin 13.

## 3 CURRENT ADVANCES IN CYTOKINE DETECTION PLATFORMS

### 3.1 Methods for cytokine detection in clinic practice

Cytokines are ubiquitous molecules being widely present in the different body fluids, such as blood, interstitial fluids (ISF), cerebrospinal fluids (CSF), saliva, sweat, tears, gut, urine, and stool. Cytokine levels in the serum of healthy people are in pM range.^[^ ^67^ ^]^ Cytokines dysregulate, and the diseases occur. It is challenging to detect cytokines due to the low concentration in vivo instability, dynamic secretion process and complex cytokine networks.^[^ ^8^, ^68^ ^]^ The most popular methods for quantifying cytokines in clinical practice are immunoassays including enzyme‐linked immunosorbent spot (ELISpot) and enzyme‐linked immunosorbent assay (ELISA).^[^ ^69^ ^]^ Multiple steps of loading antibodies and samples in immunoassaysmake the detection tedious and time‐consuming. With the development of bioassays, flow cytometry, Luminex bead‐based assays, and the electrochemiluminescent multiplex immunoassays (Meso Scale Discovery, MSD) have achieved great success in detection of multiple cytokines in serum and plasma samples by using either multiple fluorescent beads‐based coding or physically isolated spots‐based spatial coding.^[^ ^70^ ^]^ These methods are highly sensitive and have the multiplexing capability, but they are expensive and still time‐consuming, and require complicated sample preparation, centralized instruments, and trained personnel. Moreover, special attention needs to be paid at method standardization when comparing results of cytokines between different labs in clinical studies.^[^ ^71^ ^]^ Huge discrepancies exist when samples are measured under different conditions. Factors such as cytokine binding proteins, variable cytokine forms, and interferences in matrix samples, affecting accuracy and specificity of cytokine assays were previously discussed.^[^ ^72^ ^]^ In addition, cytokine stability and clinical sample handling such as freeze and thaw cycles has a huge impact on the accuracy of cytokine detection.^[^ ^73^, ^74^, ^75^ ^]^ Notably, neither of these widely used methods is suitable for rapid cytokine monitoring within a small sample volume at the point‐of‐need, which is critical for monitoring immune status after infection or drug treatment.

### 3.2 Biosensors for detection of cytokines

**FIGURE 1:**
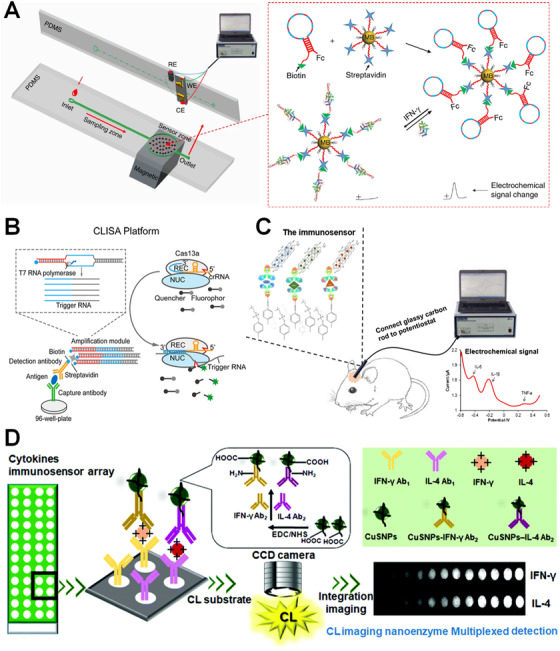
(A) A structure‐switching aptamer‐based biosensor for real‐time detection of cytokine IFN‐γ in serum. Reproduced with permission from Ref. [94]. Copyright 2019, Springer Nature. (B) A CRISPR/Cas‐based biosensing platforms for detection cytokines. Reproduced with permission from Ref. [102]. Copyright 2020, American Chemical Society. (C) An electrochemical sandwich immunosensor for simultaneous detection of three cytokines in vivo for early diagnosis of Parkinson's disease. Reproduced with permission from Ref. [105]. Copyright 2020, Elsevier. (D) A nanozyme‐based immunosensor for detection of multiple cytokines. Reproduced with permission from Ref. [110]. Copyright 2018, the Royal Society of Chemistry

Cytokines form a very complex cytokine network, which mediates our immune system. Considering their critical significance in understanding of human health and diseases, researchers are working around the clock to develop tools for cytokine detection aiming to make a breakthrough in sensitivity and multiplex detection capability.^[^ ^76^, ^77^ ^]^ Liu et al. have developed different biosensing platforms for single cytokine monitoring from in vitro to in vivo with fluorescence signal readout ^[^ ^78^, ^79^, ^80^, ^81^, ^82^, ^83^, ^84^ ^]^ or electrochemical signal readout.^[^ ^85^, ^86^ ^]^ In order to realize the cytokine monitoring in mouse brain or spinal cords, deployable devices based on immunosensors on optical fiber^[^ ^87^, ^88^ ^]^ and stainless steel^[^ ^80^, ^89^ ^]^ have been developed for detection of spatially localized cytokines at the levels of pg/mL. An impedance aptasensor was developed for highly sensitive and selective detection of IL‐6 with a good linear response from 5 pg/mL to 100 ng/mL and a detection limit of 1.6 pg/mL.^[^ ^90^ ^]^ The biosensor was successfully used to detect IL‐6 in blood samples collected from patients suffering of colorectal cancer with desirable performance. With the demand for multiplexing capability, shorter analysis time, smaller sample volume, and higher sensitivity,^[^ ^8^ ^]^ in addition, how to realize the detection platforms to realize real‐time cytokine monitoring is the bottleneck problem of cytokine biosensing.^[^ ^91^ ^]^ With the capability of switching the 3D configuration with the presence of the target analyte, structure‐switching aptamers have demonstrated as excellent recognition unit for continuous cytokine monitoring.^[^ ^92^, ^93^, ^94^, ^95^, ^96^ ^]^ An electrochemical biosensor based on structure‐switching aptamers against to IFN‐γ was developed for the successful detection of IFN‐γ continuously (Figure 1A). Ideally, a noninvasive way that differs from the conventional brain implantable biosensor,^[^ ^97^ ^]^ needs to be explored and the wearable electronics for sweat derived cytokines detection might be a potential direction.^[^ ^98^, ^99^ ^]^ The current main challenge is the continuous detection of multiple cytokines in vivo without background drift and the interferences in matrix samples.^[^ ^100^, ^101^ ^]^

CRISPR/Cas biosensing system has demonstrated its success in cytokine detection with superior sensitivity, the limit of detection (LoD) values for human IL‐6 and human VEGF are 45.81 fg/mL and 32.27 fg/mL, respectively (Figure 1B).^[^ ^102^ ^]^ It should be noted that simultaneous detection of multiple cytokines is far more informative than that from one single cytokine using singleplexed detection.^[^ ^103^ ^]^ To realize electrochemical detection of multiple cytokines (IL‐6, IL‐1β, and TNF‐α) in serum, a sandwich immunosensor was developed. The multiplexing was realized by using three distinct redox probes, that is, nile blue (NB), methyl blue (MB), and ferrocene (Fc), for labeling different detection antibodies and signals were amplified by loading numerous redox probes to the graphene oxide. The assay demonstrated the similar results to ELISA but with fast assay time (< 30 minutes) and the superior capability for multiple cytokine detection (Figure 1C).^[^ ^104^ ^]^ In the follow‐up study, it was observed that levels of these three cytokines are elevated about 5 times in a Parkinson's disease mice model comparing to the control groups.^[^ ^105^ ^]^ Instead of using external multiple reporters for labeling/coding to allow the multiplexing, Lau et al. utilized spatial coding strategy by using electron beam lithography for the direct immobilization of anticytokine capture antibodies on different spots of silicon substrates using a trehalose glycopolymer as a resist.^[^ ^106^ ^]^ The target analytes can thus recognize the spatially resolved capture antibodies, and patterns can be detected using dark‐field microscopy after addition of silver‐enhanced gold nanoparticle conjugated detection antibodies. This sandwich immunoassay was able to do simultaneous detection of IL‐6, and TNF‐α secreted from stimulated RAW 264.7 macrophages. The direct fabrication of capture antibody patterns on chips for cytokine detection is potential for preparation of printed biosensors. Recently, a sensitive surface‐enhanced Raman scattering (SERS) nanotags‐based detection platform was developed for detection of cytokine TNF‐α secreted by Lymphoma cells with the sensitivity of 4.5 pg/mL,^[^ ^107^ ^]^ which demonstrated the proof‐of‐concept for detection of three cytokines, IFN‐γ, TNF‐α, and IL‐10 secreted from the lymphoma cell lines upon the Con A stimulation although no calibration curve was generated. However, for immunosensor‐based cytokine multiplexing assays, cross‐reactivity can compromise their performance by providing decreased sensitivity, increased variability.^[^ ^108^ ^]^ Singleplex based on a microfluidic technology was designed to eliminate cross‐reactivity between competing analytes allowing rapid and sensitive detection of multiple cytokines and chemokines from human and mouse samples.^[^ ^109^ ^]^ Another sensitive chemiluminescence (ECL) nanozyme immunoassay was developed for simultaneous detection of two chicken cytokines (IL‐4 and IFN‐γ) in serum samples in the range of 0.01‐60 ng/mL for IFN‐γ and IL‐4, respectively and the detection limits of IFN‐γ (2.9 pg/mL) and IL‐4 (3.2 pg/mL) (Figure 1D).^[^ ^110^ ^]^ In the system, capture antibodies were coated on an epoxy silane coated glass array containing 4 × 12 spots fabricated by screen‐printing. After incubation with samples containing cytokines, CuSNPs‐based nanozyme tags were used to label with secondary antibodies. Thus, a typical dot blot sandwich assay was set up, and the ECL signal on each spot can be captured using CCD camera upon addition of luminol‐H_2_O_2_ substrates. The spatially resolved multiplexing platform overperformed the conventional HRP enzyme labels with higher sensitivity, higher throughput, low cost, reduced consumption, more rapid assay speed and easier operation. This work opens a promising avenue for the exploitation of novel and universal nanozyme labels for high‐throughput and sensitive multiplex detection of cytokines. Although these stated examples in Section 3.2 present the high sensitivity and multiplexing capability of cytokines in complex biological fluids such as blood, CSF samples, multiple steps of manual manipulation are needed which requires tedious workload and inevitably increase the risk of variation between detections using the same sensing platform. Therefore, it is crucial to develop bioanalytical platforms in POC settings to realize on‐site cytokine detection without the need of additional equipment or tedious preparation work, achieving a “sample‐in‐result‐out” real‐time disease management and immunology studies.

## 4 ADVANCES IN POC DETECTION PLATFORMS FOR CYTOKINES

### 4.1 Basics of POC detection

Driven by inspiring clinical correlation of cytokines with diseases (Sections 2.1 and 2.2) and the very recent situation in health care delivery caused by current COVID‐19 pandemic, POC measurements continue to be in unmet demand because POC tests can provide rapid answers while significantly reducing the analysis procedure, personnel and cost.^[^ ^111^, ^112^ ^]^ Encouragingly, cytokine detection in a POC fashion is in the infancy but rapid expansion stage. POC testing devices usually include two major categories: (1) small handheld or wearable devices such as glucose test strips, continuous glucose monitor (CGM), and pregnancy test strips, providing qualitative or quantitative determination of an increasing range of analytes and (2) bench‐top but portable devices such as oximeter, small hematology, and immunology analyzers, which are lab based but in small size and simplicity.^[^ ^113^ ^]^ This review focuses on the first category covering the main formats of paper lateral flow assay (LFA), electrochemical microfluidic paper‐based analytical devices (μPADs), the latest field‐effect transistor (FET)‐based POC devices. As summarized in Table 2, the cytokine POC devices have achieved highly active academic developments in terms of hardware and software engineering, and their integration and miniaturization. More entries into real applications are largely accelerated with the improved sensitivity, reproducibility, and the multiplex‐detection capability.^[^ ^114^ ^]^

**TABLE 2:** Comparison of POC platforms for detection of cytokines

Target cytokine	Sensing format	Sample	Sample volume (μL)	Assay time (minute)	Detection limit (pg/mL)	Dynamic range (pg/mL)	Ref.
IL‐6	colorimetric LFA	Plasma	150	20	380	1250‐9 × 10^6^	[115]
IL‐6	fluorescence LFA	Serum	70	15	0.37	2‐500	[116]
IL‐10, IFN‐γ	fluorescence LFA	Serum	10	15	30	30‐1000	[144]
IL‐6	fluorescence LFA	Blood	10‐50	6	0.002	0‐0.84	[118]
IL‐6	colorimetric LFA	Buffer	40	<1	29	0.1‐10	[145]
IL‐6	colorimetric LFA	Blood	2.5	17	0.1	0.001‐10	[146]
IL‐6	fluorescence LFA	Serum	–	–	0.9	1‐1000	[147]
IL‐1β, IL‐12p70, TNFα	Fluorescence	Serum	10	<15	7.4 (IL‐1β), 2 (IL‐12p70), 6.5 (TNFα)	60‐150	[148]
IL‐6, IL‐8, IL‐10, TRAIL, IP‐10	EIS	Plasma	∼40	∼5	0.1 (IL‐6), 0.1 (IL‐8), 1 (IL‐10), 1 (TRAIL), 1 (IP‐10)	0.01‐10 000 (IL‐6), 0.01‐5000 (IL‐8), 0.1‐1000 (IL‐10), 1‐1000 (TRAIL), 1‐2000 (IP‐10)	[131]
TGF‐β1	CA	Saliva	5	5	0.95	2.5‐1000	
IFN‐γ	EIS	Serum	25	30	3.4	5‐1000	[129]
IFN‐γ	EIS	PBS	–	<35	520	1000‐5000	[149]
IFN‐γ, IL‐10	EIS	Serum	–	–	25 (IFN‐γ), 46 (IL‐10)	100‐5000 (IFN‐γ), 100‐2000 (IL‐10)	[150]
IL‐3	CA	Blood	100	<60	∼5	1‐1000	[11]
IL‐β1, TNF‐α	EC	Serum, saliva	1.5	150	0.38 (IL‐1β), 0.85 (TNF‐α)	0.5‐100 (IL‐1β), 1‐200 (TNF‐α)	[151]
CEA, NSE	DPV	Serum	20	–	2 (CEA), 10 (NSE)	10‐5 × 10^5^ (CEA),	
						5‐5 × 10^5^ (NSE)	[152]
IL‐4	FET	CCM	20	Real time	2.5	0.025‐2.5 × 10^6^	[136]
IL‐6	FET	Saliva	10	400	286	1.19×10^3^‐2.38 × 10^3^	[137]
TNF‐α	FET	Sweat	–	5	456	877‐8.77 × 10^6^	[138]
TNF‐α	FET	Sweat	–	–	87.7	877‐1.75 × 10^6^	[139]
IFN‐γ	FET	Sweat	–	–	11.8	240‐4 × 10^6^	[141]
IFN‐γ	CA	Plasma	100	8	40	16‐2048	[153]

IL‐6: interleukin 6, TRAIL: tumor necrosis factor‐related apoptosis‐inducing ligand, IP‐10: interferon gamma‐induced protein‐10, HC: healthy control, TGF‐β1: transforming growth factor β1, IFN‐γ: interferon‐gamma, CCL4: chemokine CC motif ligand 4, IP‐10: IFN‐γ‐inducible protein 10, LFA: lateral flow assay, VL: visceral leishmaniasis, CCM: cell culture media, DPV: differential pulse voltammetry, CA: chronoamperometry, CEA: carcinoembryonic antigen, NSE: neuron‐specific enolase, FET: filed effect transistor, EIS: electrochemical impedance spectroscopy.

### 4.2 Paper lateral flow assay‐based optical POC detection

**FIGURE 2:**
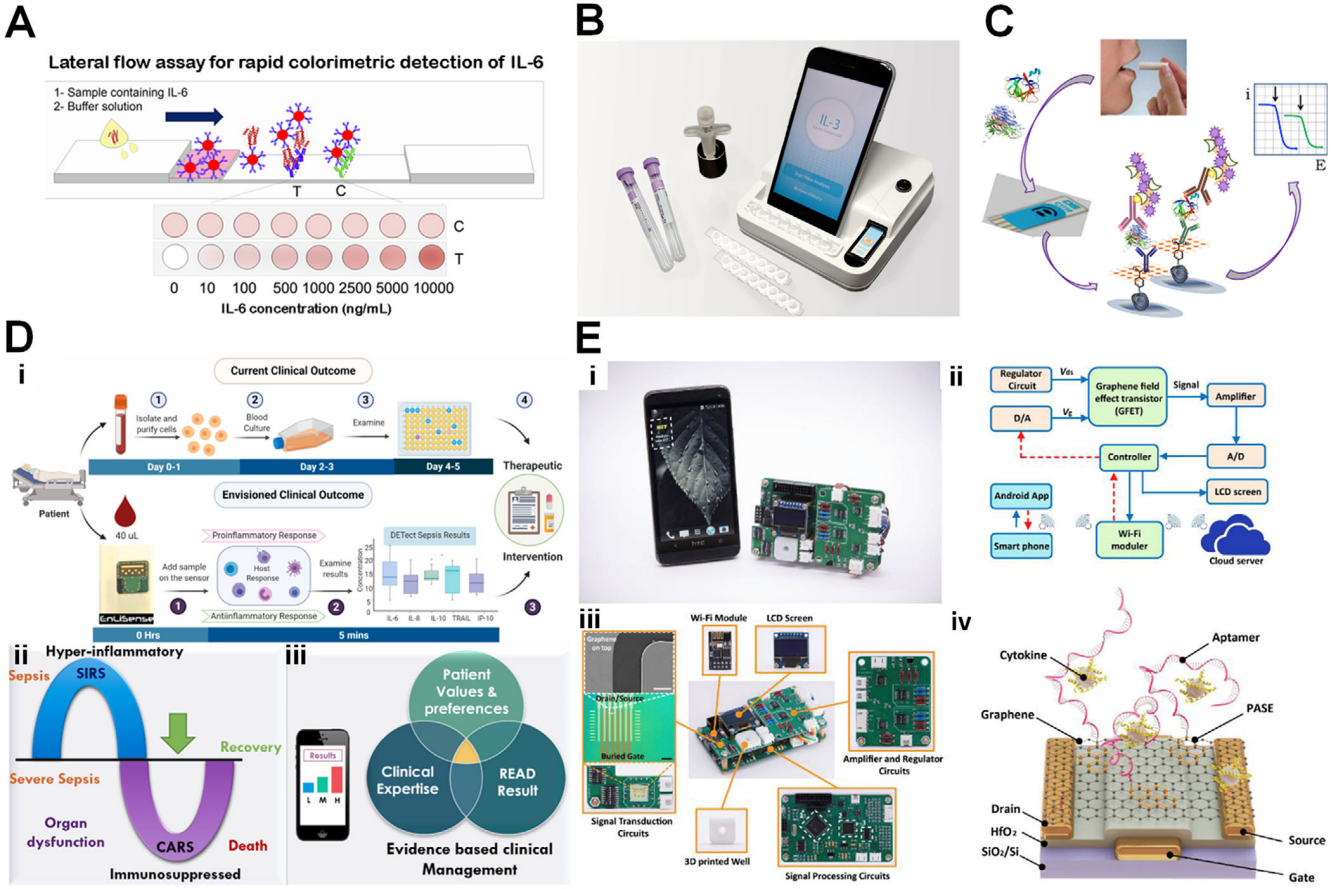
(A) A paper lateral flow assay for POC detection of IL‐6. Reproduced with permission from Ref. [115]. Copyright 2020, Maples Scientific Publishers. (B) A hybrid magneto‐electrochemical sensor for rapid and sensitive detection of IL‐3 for early diagnosis of sepsis. Reproduced with permission from Ref. [11]. Copyright 2018, American Chemical Society. (C) The screen‐printed electrode‐based electrochemical immunosensor for simultaneous determination of IL‐β1 and TNF‐α. Reproduced with permission from Ref. [130]. Copyright 2017, Elsevier. (D) An electrochemical POC sensing device for multiplexed cytokine detection toward rapid sepsis endotyping. Reproduced with permission from Ref. [143]. Copyright 2020, Elsevier. (E) An integrated system based on field effect transistor was developed for online detection of IL‐6. Reproduced with permission from Ref. [137]. Copyright 2019 Elsevier

Paper‐based analytical devices represent the majority portion of POC devices because paper is a biocompatible and low‐cost substrate with high feasibility to integrate different function modules, favoring its use in diagnostics of biological samples. LFA using porous membrane is one of the most successful formats for POC detection. For a typical sandwich LFA, the target cytokine in a biofluid sample can bind to the detection probe (recognition molecule, such as the most popular antibody decorated gold nanpparticles nanoparticles [AuNPs]) to form nanocomplex, which can migrate through, for example, nitrocellulose membrane, recognize and bind to the test line pre‐embedded with capture probes (e.g., secondary antibody), displaying a red indication line. The excess detection probe without target can be recognized by the secondary antibody thus exhibit another red indication line as the control. For example, AuNPs‐based LFA was developed for rapid and colorimetric IL‐6 detection using plasma samples of patients with severe visceral leishmaniasis (VL). The developed LFA assay time takes 20 minutes with a linear range of 1.25‐9,000 ng/mL and a detection limit of 0.38 ng/mL (Figure 2A).^[^ ^115^ ^]^ Besides the AuNPs, other optical colloids can be also employed as signal readout. Huang et al. reported a double‐antibody sandwich immunofluorescent LFA using europium nanoparticles as signal tag was developed for rapid quantitative detection of IL‐6 in serum samples from septic patients. A wide linear range (2‐500 pg/mL) with a good sensitivity (0.37 pg/mL) and the assay time (∼15 minutes) was achieved,^[^ ^116^ ^]^ with a high correlation (*n* = 214, *r* = 0.9756, *p *< 0.01) to the commercial SIEMENS CLIA IL‐6 kit. As the singleplexed assay offer limited information of one cytokine, multiplexed LFA assay were investigated aiming to provide more comprehensive information. Usually this can be realized by development of multitest lines, that is, modification of the membrane with physically separated multiple capture antibodies targeting different cytokines. In such regard, Paul et al. explored simultaneous detection of IL‐10 and IFN‐γ utilizing the lanthanide‐based upconverting phosphor nanoparticles conjugated with corresponding antibodies as reporters.^[^ ^117^ ^]^ The assay was used to evaluate blood samples of leprosy patients and demonstrated a quantitative correlation value of 0.92 compared with commercial ELISA. Besides the spatially resolved multitest line‐based multiplexing, multiple reporter labeling is another common approach. For example, an optical duplex immune‐LFA was fabricated using green and red quantum dots (same excitation wavelength but different emission wavelengths) as labels for two antibodies targeting C‐reactive protein (CRP) and IL‐6.^[^ ^118^ ^]^ The simultaneous quantification of CRP and IL‐6 in a single test line was realized by using a single UV‐light source and two suitable emission filters for readout through a widely available BioImager device. A customized software tool, the MultiFlow‐Shiny app was used to accelerate and simplify the readout process, which were superior to the popular software ImageJ and resulted in low detection limit of 2 fg/mL for IL‐6. This assay may serve as a powerful tool for POC diagnosis of inflammation and infectious events. Besides the common LFA format with colorimetric and fluorescent detection, with benefits from the natural plasmonic property of noble metal nanoparticles and their compatibility with LFA, LFA, with SERS signal read‐out was also explored for cytokine POC detection. For example, Thomas et al. developed Au (50 nm)/Au (17 nm) core/satellite‐based SERS‐active tags labeled with the corresponding antibodies for detection of IL‐1β and IFN‐γ using LFA.^[^ ^119^ ^]^ With the development of portable Raman spectrometer,^[^ ^120^ ^]^ the on‐site SERS‐LFA diagnostic platforms have been realized for infectious diseases early diagnosis with enhanced sensitivity.^[^ ^121^ ^]^ It should be noted that the repeatability associated with LFA for cytokines is still the critical factor to limit its wide applications in clinic. This might be solved with high‐quality control of biocomponents, fabrication process, and automation to minimize the variations.

### 4.3 Electrochemical paper‐based POC devices

Since the first paper‐based microfluidic was discovered as a promising analytical platform by Whiteside's research group in 2007,^[^ ^122^ ^]^ microfluidic paper‐based analytical devices (μPADs) are emerging as promising lightweight, disposable, and cost‐effective formats for developing POC testing.^[^ ^123^ ^]^ Electrochemical μPADs, mostly fabricated by screen‐printing technology, stand out another prototype of paper‐based POC device, offering a sensitive, specific, and miniaturized platforms, and have been extensively explored recently.^[^ ^124^ ^]^ Paper plays a role of support substrate for the electrodes and also the matrix where sample and recognition biomolecules are joined and react. In fact, paper offers a thin, mechanically stabilized film of water, or other fluids, that deliver analytes to the surface of the electrodes.^[^ ^125^, ^126^ ^]^ The most widely used paper substrate to date is the Whatman grade 1 chromatographic filter paper. Recently, an amperometric sandwich immunosensor fabricated on a screen‐printed electrode (SPE) was developed for the determination of the clinically relevant endogenous cytokine IFN‐γ in saliva.^[^ ^127^ ^]^ The sensing interface was firstly modified with p‐aminobenzoic acid by the diazonium salt chemistry followed by fabrication of a specific capture antibody. The biotinylated antibody labeled with a streptavidin‐horseradish peroxidase conjugate was used as the signal reporter. The developed method has a linear range of 2.5‐2000 pg/mL and a detection limit of 1.6 pg/mL, and was comparable to a commercial ELISA kit. A similar electrochemical immunosensor was fabricated on SPE for detection of transforming growth factor β1 (TGF‐β1) in saliva.^[^ ^128^ ^]^ In order to enhance the sensitivity, the signal tags based on single‐walled carbon nanotubes were labeled with viologen, horse radish peroxidase, and anti‐TGF antibodies. The analytical characteristics for detection of TGF‐β1 (a linear range of 2.5‐1000 pg/mL; a detection limit of 0.95 pg/mL) was improved notably comparing to other reported immunosensors or ELISA kits.

By stepping further, an electrochemical μPADs was developed using wax‐printing technique for sensitive impedance detection of human IFN‐γ.^[^ ^129^ ^]^ A linear relationship between impedance and logarithmic concentrations of human IFN‐γ in serum was found in a range of 5‐1000 pg/mL with a detection limit of 3.4 pg/mL. Polyaniline‐graphene modified SPE provided 31‐fold higher sensitivity compared to polyaniline modified electrodes. This system is rapid, cost effective, and disposable, allowing the POC screening of IFN‐γ in biological samples. A more advanced POC detection platform based on a hydrid hybrid magneto‐electrochemical sensor was developed for rapid (with 1 hour) sensitive (< 10 pg/mL) detection of IL‐3 for early diagnosis of sepsis (Figure 2B).^[^ ^11^ ^]^ The electrochemical signal corresponding to the analyte concentration was converted to the electric signal by this POC station. The analyte concentration was finally reported by the smartphone app and uploaded to a cloud sever via Bluetooth. This sensing platform was successfully used to detect IL‐3 in blood from people with sepsis and was 5 times faster and 10 times more sensitive than conventional ELISA. This smart POC detection system could be a practical tool for timely diagnosis and prevention of sepsis in clinic although the assay time needs to be further reduced. The SPE‐based electrochemical immunosensor with amperometric signal amplification was developed for simultaneous determination of IL‐β1 and TNF‐α in human serum spiked at clinically relevant concentration levels and in real saliva samples (Figure 2C).^[^ ^130^ ^]^ Under optimized conditions, the dual immunosensor allows ranges of linearity extending between 0.5 and 100 pg/mL and from 1 to 200 pg/mL for IL‐1β and TNF‐α, respectively, which cover cytokine levels in clinical samples. The achieved detection limits were 0.38 pg/mL (IL‐1β) and 0.85 pg/mL (TNF‐α), respectively. In addition, the dual immunosensor exhibits excellent reproducibility of the measurements and storage stability. Recently, a novel immunosensor‐based POC device was designed to monitor a panel of five cytokines (IL‐6, IL‐8, IL‐10, TRAIL & IP‐10), the potential biomarkers for sepsis with high sensitivity (a detection limit of ∼1 pg/mL), short assay time (< 5 minutes, ∼30 times faster compared to the standard reference technique), and small sample volume (a single drop of undiluted plasma sample).^[^ ^131^ ^]^ The concentration of target biomarkers can be monitored simultaneously using nonfaradaic electrochemical impedance spectroscopy (Figure 2D). This work provides a technology for effective clinical management of sepsis at the patient bedside. Electrochemical biosensors (especially μPADs) have demonstrated their potentials for POC detection of cytokines. With sensitive and reliable cytokine assays, development of portable devices being capable to converting electrochemical signal associated with cytokine concentration to electric signal will continue to govern the success of electrochemical μPADs in cytokine POC detection. It is worthy to note that the electrochemical paper‐based devices offer cheaper and faster test platform and their operation does not require highly trained personnel, which is clear advantageous over the conventional ELISA. The major concern and challenge associated with its applications is the preserve the activity of biocomponents stored in the pores of the paper device since the enzymes and antibodies, which may be prone to oxidation by air. Therefore, it is highly favorable to develop appropriate sealing or packing technologies, for example, polybags or metal‐organic frame‐based biomineralization that enables storage of paper‐based biosensors in dry atmosphere without degradation of biomolecules.^[^ ^132^, ^133^, ^134^ ^]^

### 4.4 Field‐effect transistor‐based POC detection and others

Beside the common prototype of LFA and electrochemical sensor‐based cytokine POC device, field‐effect transistors (FET) have also attracted dramatic attention in the field of cytokine rapid test. In a FET sensing device, the nonmetalized gate dielectrics that are exposed to an electrolyte solution covering the underlying semiconductor material actively transduce the biological binding events on the surface. The efficiency of FET‐based novel devices for detection of different cytokine analytes in a real time, highly precise manner has been explored by a number of studies.^[^ ^135^ ^]^ For example, one‐dimensional ion‐sensitive FET arrays (nanoISFETs) on silicon nanowire were fabricated for continuous POC detection of cytokines (IL‐4 and IL‐2) secreted in mouse T helper cell differentiation culture media.^[^ ^136^ ^]^ Such portable sensing platform was able to detect IL‐4 concentrations with a broad dynamic range between 25 fg/mL (1.92 fM) and 2.5 μg/mL (192 nM) with a detection limit down to 35 fM, indicating a highly adaptive platform for human cytokine POC test. In another study, Zhao and coworker developed a graphene‐based fully integrated portable FET sensing system for online detection of IL‐6 within 400 seconds in saliva with a detection limit down to 12 pM (Figure 2E).^[^ ^137^ ^]^ The authors integrated the FET aptasensor and online signal processing circuits on printed circuit boards (PCBs). Specifically, this miniaturized system used a buried‐gate geometry with HfO_2 _as the dielectric layer and online signal processing circuits to realize the transduction and processing of signals which reflect cytokine concentrations. The signal can be wirelessly transmitted to a smart‐phone or cloud sever through the Wi‐Fi connection for visualizing the trend of the cytokine concentration change, offering the practicality for noninvasive saliva diagnosis of diseases at early stage. Beneficial from the merits of online signal processing using the integrated FET sensor, the same group further extended the applications of the graphene‐based FET sensor for wearable detection of TNF‐α^[^ ^138^, ^139^ ^]^ and IFN‐γ^[^ ^140^, ^141^ ^]^ in human sweat. Additionally, a handheld saliva swab‐to‐result platform was developed for detection of HIV antibodies and TNF‐γ by a combination of a novel nanopore assay, a portable reader device and a disposable test strip within 60 seconds.^[^ ^142^ ^]^ FET sensors have advantages in realizing real‐time detection with high sensitivity, contributing to the cytokine POC detection. With the constant enhancement of nanotechnology and improvement of readout systems, the performance of FET biosensing platforms were further improved. However, how to improve the specificity in complex bodily fluids and the possibility for high‐throughput analysis and multiplexing capability still require further investigations.

## 5 CHALLENGES AND PERSPECTIVES FOR POC DETECTION OF CYTOKINES

**FIGURE 3:**
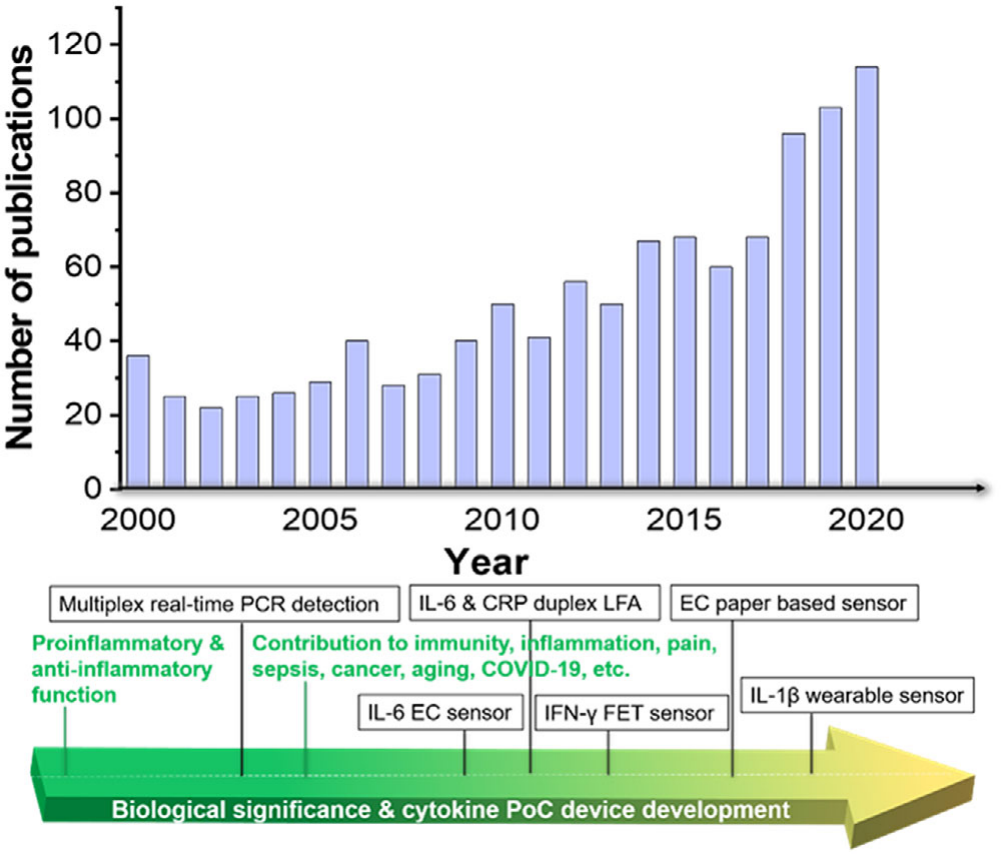
Overview of developments in the field of cytokine POC devices. The histogram displays the number of publications/year using the search terms (“portable” OR “point of care” OR “paper” OR “strip” OR “lateral flow” OR “disposable” OR “wearable”) AND (“cytokine”) as analyzed by Web of Science. This search found 1075 articles published with these keywords (since 2000) with a total of 28,190 citations. Displayed underneath is an approximate timeline of the most related discoveries in the context of disease related cytokine development and selected milestone cytokine POC devices. The proinflammatory role of cytokines,^[^ ^154^ ^]^ multiplex real‐time PCR detection of cytokines,^[^ ^155^ ^]^ cytokine‐mediated link between innate immunity, inflammation, and cancer,^[^ ^156^ ^]^ IL‐6 electrochemical sensor,^[^ ^157^ ^]^ IL‐6 and CRP duplex LFA,^[^ ^158^ ^]^ IFN‐γ FET sensor,^[^ ^159^ ^]^ electrochemical paper‐based cytokine biosensor^[^ ^125^ ^]^ IL‐1β, and CRP wearable sensor^[^ ^160^ ^]^

The last two decades have witnessed intensive study of cytokine biological role in numerous diseases, and the rapid development of relevant POC devices in parallel (Figure 3), which have been systematically discussed in this review. Cytokine storm and cytokine POC devices have received unprecedented attention during the special year with the outbreak of COVID‐19, which will undoubtedly arouse broader interest of human community in disease management of cytokine related medicine and research and industry development of the POC devices. It is expected to the future research efforts in the field of cytokine POC detection would be in line with the following aspects.

### 5.1 Sensitivity

**FIGURE 4:**
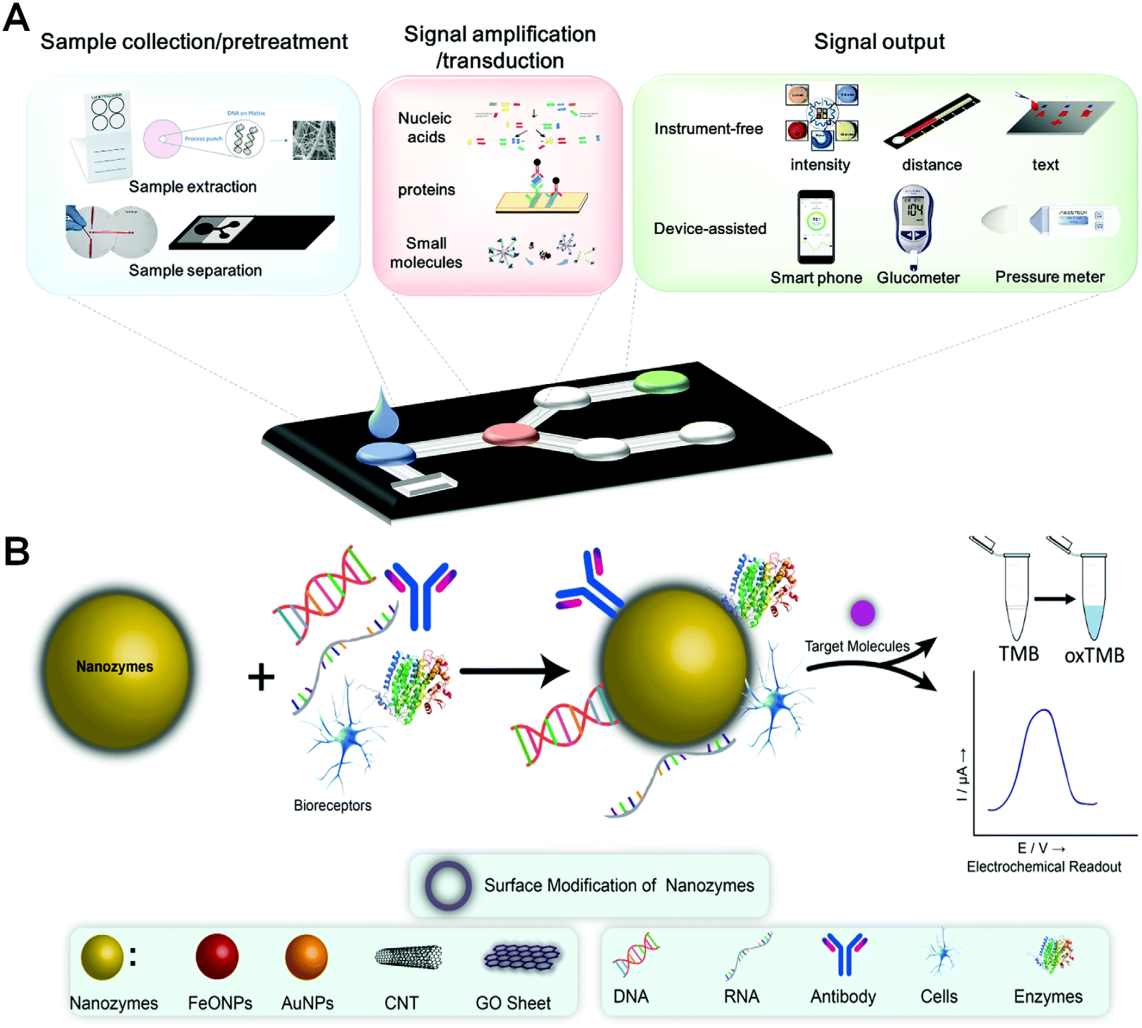
(A) A sample‐in‐answer‐out μPAD is expected to enable sample collection/pretreatment, signal amplification/transduction and signal output. Reproduced with permission from Ref. [162]. Copyright 2018, Royal society of chemistry. (B) Schematic representation of the nanozyme's catalytic activities and its application in the electrochemical biosensor. Reproduced with permission from Ref. [166]. Copyright 2020, Royal society of chemistry

Sensitivity is always one of the most important factors for developing a successful biosensor. Ultrasensitivity (typically < 1 pM) would endow the sensory device with capability of detecting cytokine at ultra‐low level, that is, detectable and distinguishable signal from background noise. This would permit reliable detection using only small input volume of biofluids even without the need of analyte enrichment. POCT aims to provide simple, fast, and near‐of‐need detection, and they require no or limited sample treatment, the minimum sample volume, limited signal amplification, etc. Thus, compared to other diagnostic methods, POCT has relatively low detection sensitivity. Cytokines are low abundant proteins in our body and the cytokine leaves are in low pM range under healthy conditions, which makes sensitivity is extremely important for cytokine detection, and also one of the most significant challenges associated with POC cytokine detection in early detection of infectious diseases, or cancer. Recently, Liu and Yang group specifically reviewed strategies on enhancing sensitivity of μPADs (Figure 4A),^[^ ^161^, ^162^ ^]^ including (1) nanomaterials‐based signal amplification, which is because nanomaterials have the high surface to volume ratio and versatile surface chemistry helping to fabricate maximum amount of recognition molecules or signal tags^[^ ^163^, ^164^, ^165^ ^]^ would be beneficial for higher sensitivity. Nanozymes, enzyme‐mimetic nanomaterials are the recent superstars in the field of molecular diagnostics (Figure 4B).^[^ ^166^ ^]^ Nanozymes are having the advantages such as high stability, low cost, and versatile capability in catalyzing reactions with enhanced speed and sensitivity. These characteristics greatly are beneficial for their wide application in POC detection by enhancing the sensitivity and integrating with the whole analysis system to realize the smart cytokine detection.^[^ ^110^, ^167^ ^]^ (2) Nucleic acid‐based signal amplification. Polymerase chain reaction (PCR) is the extensively used as signal amplification techniques for detection of nucleic acids or other analytes, which can be recognized by aptamers or antibody‐DNA conjugate. Normally, thermal cycling is required in PCR, which limits their applications in POC detection.^[^ ^162^ ^]^ Fortunately, lots of isothermal nucleic acid amplification techniques,^[^ ^168^ ^]^ such as recombinase polymerase amplification, loop‐mediated isothermal amplification (LAMP),^[^ ^169^, ^170^ ^]^ rolling circle amplification (RCA), strand‐displacement amplification, and so on are widely used to amplify the signal of a bioassay and offer on‐site detection of various targets.^[^ ^171^ ^]^ These methodologies can be readily adapted to cytokine detection with the conversion of cytokine binding signal via nucleic acid signal in an amplified manner. Since 2017, clustered regularly interspaced short palindromic repeats/Cas enzymes (CRISPR/Cas)‐based biosensors have attracted wide attention due to the high sensitivity and specificity.^[^ ^172^ ^]^ Being integrated with ELISA, CRISPR/Cas was successfully applied for the detection of multiple cytokines with the fetomolar sensitivity.^[^ ^102^ ^]^ CRISPR/Cas biosensing system has demonstrated great potentials in POC detection.^[^ ^173^ ^]^ (3) Device engineering‐based signal amplification. In additional to the chemical methods for signal amplification, engineering the POC devices is also helpful to enhance sensitivity. For example, In order to increase the sensitivity of conventional LFA, a new LFA design based on geometric flow control was reported.^[^ ^145^ ^]^ This novel approach enables comprehensive flow control *via* different membrane geometric features such as the width and the length of a constriction, as well as its input angle and output angle. The geometric flow control lateral flow immunoassay devices (GFC‐LFID) attained a 10‐fold increase in sensitivity for detection of IL‐6 over a linear range of 0.1‐10 ng/mL with a limit of detection (LoD) of 29 pg/mL. Compared with conventional LFA, the new developed GFC‐LFA is superior in scalable fabrication process, tailored flow control, improved analytical performance, and reduced antibodies consumption (10‐fold less).

### 5.2 Multiplex‐ability

The multiplexing assay could save time and sample input, and reduce variations of multiple singleplexed assays. Especially, multiplexed POCT plays a pivotal role in clinical practice.^[^ ^174^ ^]^ Considering the biological significance of cytokines, monitoring of multiple cytokines would provide more comprehensive mapping of disease feature and more precise biological signature than the single cytokine detection. The detection platforms based on large‐scale instruments with sensing arrays take the main role in the high‐through multiplexing; for example, the commercial R&D cytokine array kit can screen more than 105 cytokines simultaneously. To date, most POC devices suffered limited multiplexing ability (only with capability of detecting < 10 cytokines at the moment) because of lots of challenges such as limited signal readout, technical variation between different labs, cross‐reactivity effect, etc.^[^ ^175^ ^]^ For example, the most popular format used in POC is the LFA with multitest lines.^[^ ^176^, ^177^, ^178^ ^]^ However, multiplexing in LFAs is a challenge due to the confined small sensing domain and thus the limited sensitivity. Combining with advances in dispensing technologies and assay development, a LFA was able to detect seven analytes in a single test strip by dispensing picolitre sensor on the sensing zone to achieve molecular encoding of analytes.^[^ ^179^ ^]^ Although there are few high‐throughput POC cytokine devices, with the aid of assay development, it is possible to realize the robust high throughout cytokine POC devices by integrating with the advances in the field of micro/nanofabrication, 3D printing, printed circuit board that could realize high integration and coding of a panel of detection probes into an small array.^[^ ^180^ ^]^ Combining with digital microfluidics, we are expecting more smart portable devices will developed for POC detection of multiple cytokines simultaneously.^[^ ^181^ ^]^ Lots of opportunities for multiplexed POC analysis of cytokines are ahead, in particular from the perspective of machine learning and deep‐learning aimed at identification of predictive biological signatures.^[^ ^182^ ^]^

### 5.3 Capability in clinic diagnostics

There are several challenges associated with POC devices for cytokine detection. Matrix effect in clinical samples is the most important challenge for POC device in clinic practice.^[^ ^183^, ^184^ ^]^ From the assay technology aspect, considering the low level of cytokines and the abundant biofoulings coexisting within the biological fluids, it is necessary to include some “bonus” units to the POC device, for example, strategies of designing an biosensor with antifouling capability for in vitro and in vivo application using PEG or zwitterion chemistry aiming to largely reduce nonspecific adsorption and enhance the signal‐to‐noise.^[^ ^101^, ^185^ ^]^ For continuous cytokine screening in vivo, radiometric measurement is helpful to eliminate the background drifting.^[^ ^96^ ^]^

From the biomarker discovery aspect, to set up a cytokine‐based biomarker for clinical practice, large cohort screening and cross‐cohort validation is needed. Considering the large size of cytokine family, high‐throughput screening platform can be employed to pick up the cytokine candidate with highest relevance to a specific disease. It is on right the track to develop a typical cytokine with “universal cut‐off” value based on large cohort study. Importantly, a POC device, in parallel, can then take the role for monitoring individual subject's cytokine to map the dynamics for “personalized cut‐off” and for assessment of their qualification as the routinely used clinical biomarker. Ideally, longitudinal study allows monitoring of the cytokine candidates over a long time scale, including the status before and after therapy, which is favorable for building up an accurate health management for patients.^[^ ^186^ ^]^ The comparative study of the “universal cut‐off” and “personalized cut‐off” would further enhance our capability for mapping cytokine evolution toward personalized medicine.

Additionally, methods calibration between labs is another challenge for cytokine POC detection. Reported variations between procedures and policies used by different laboratories underline the need for harmonization of tests to allow timely and reliable communication of critical results with clinical personnel responsible for patient care. Definitely, an overall consideration on the assay performance, clinic needs, and POC performance is essential to design a POC device for cytokine detection in clinic.

### 5.4 Integrated portable device toward smart POC detection in real time

Signal readout is another essential factor to be considered for developing a successful biosensing device. POC technologies aim to provide simple, rapid, and end‐user friendly information near the patients’ need, and achieving the digital signal monitoring is desirable. Bing benefit from the advanced manufacture and the digital era, the signal readout, either the optical or electrochemical intensity, can be converted into digital format, rapidly accelerating the spreading of POC devices and their linkage with our personal equipment like smart phone. For example, the printed circuit board or integrated circuit can be incorporated with POC electrochemical sensors,^[^ ^187^, ^188^ ^]^ and the miniaturized device can be plugged into our mobile phone to realize the detection with the results directly displayed in the mobile phone. Such advances would undoubtedly make the daily health management more convenient and smarter, especially in resource limited setting and also in the post COVID‐19 pandemic time for disease prevention. With that embracement of researchers from diverse backgrounds like physics, chemistry, biology, materials, electric engineering and mechanics, etc., the inspiring digital and smart POC systems would step into rapid‐expansion development. This is particularly important as POC devices can offer rapid detection but some of them use multistep of manual manipulations and cannot realize absolute sample‐in‐result‐out, which undoubtedly increases the risk of variation thus negative impact on the reproducibility. Therefore, automation and integration of multiple‐step function into miniaturized device will undoubtedly meet the demand requirement.

**FIGURE 5:**
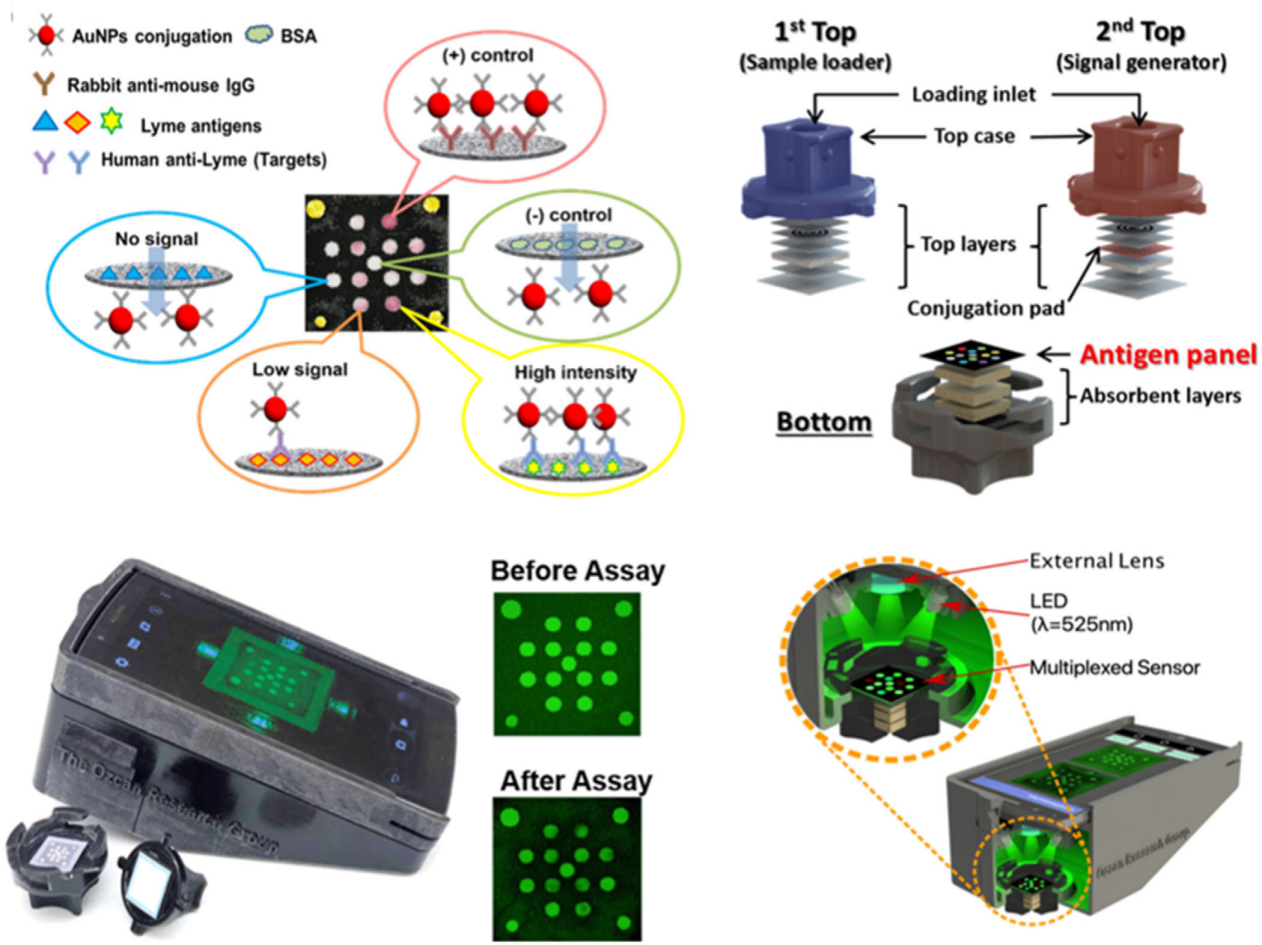
Illustration of the multiplexed immunoreactions on the sensing membrane during the vertical flow assay operation (top left). Diagram of the paper materials within the vertical flow assay showing the sample loading top case (in blue) and the signal generating top case (in red, top right). Photograph of the mobile‐phone reader with an opened vertical flow assay cassette and example images of the sensing membrane (bottom left). Cross‐section of the vertical flow assay mobile‐phone reader (inset: the sensing membrane using an optomechanical attachment, bottom right). Reproduced with permission from Ref. [189]. Copyright 2020, Royal Society of Chemistry

Apparently, adapting digital signal monitoring to POC devices to achieve a fully integrated POC devices such as wearable biosensing devices^[^ ^99^ ^]^ for continuous cytokine monitoring is challenging. It will involve the interdisciplinary knowledge in the fields of biosensors, advanced materials, electronics, software engineering, biomedical engineering, and Internet of things. Active research has been focusing on developing smartphone app or portable meters to realise mobile health (Figure 5) with capability of monitoring a panel of 20 analytes across different samples (*n* = 50),^[^ ^189^ ^]^ which clearly point out the future direction for molecular diagnostics with the convergent hardware integration (i.e., multiplexed vertical flow assay with mobile‐phone reader) and advanced algorithm (i.e., data training and validation). Such platform is highly adaptive and offers desirable way for future intelligent detection of cytokine. With the development of handheld and portable meters to read chemical signals, POC devices are able to provide rapid and sensitive quantification of cytokines in clinical samples. Additionally, with the aid of artificial intelligence and machine learning, POC technologies can enable next‐generation health care monitoring and management.^[^ ^182^, ^190^ ^]^

## CONFLICT OF INTEREST

The authors declare no conflict of interest.
